# Type-II tRNAs and Evolution of Translation Systems and the Genetic Code

**DOI:** 10.3390/ijms19103275

**Published:** 2018-10-22

**Authors:** Yunsoo Kim, Bruce Kowiatek, Kristopher Opron, Zachary F. Burton

**Affiliations:** 1 University of Michigan, Ann Arbor, MI 48109, USA; yunsoo@umich.edu; 2 Blue Ridge Community and Technical College, Martinsburg, WV 25403, USA; BKOWIATE@blueridgectc.edu; 3 Bioinformatics Core, University of Michigan, Ann Arbor, MI 48109, USA; kopron@gmail.com; 4 Department of Biochemistry and Molecular Biology, Michigan State University, E. Lansing, MI 48824, USA

**Keywords:** type-II tRNA, V loop, V loop-acceptor stem homology, minihelices, evolution of tRNA, evolution of translation systems

## Abstract

Because tRNA is the core biological intellectual property that was necessary to evolve translation systems, tRNAomes, ribosomes, aminoacyl-tRNA synthetases, and the genetic code, the evolution of tRNA is the core story in evolution of life on earth. We have previously described the evolution of type-I tRNAs. Here, we use the same model to describe the evolution of type-II tRNAs, with expanded V loops. The models are strongly supported by inspection of typical tRNA diagrams, measuring lengths of V loop expansions, and analyzing the homology of V loop sequences to tRNA acceptor stems. Models for tRNA evolution provide a pathway for the inanimate-to-animate transition and for the evolution of translation systems, the genetic code, and cellular life.

## 1. Introduction

**Figure 1:**
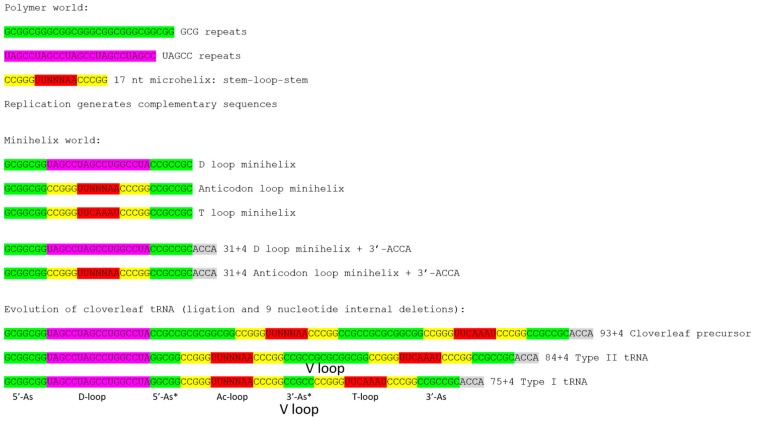
Models for the evolution of type-I and type-II tRNAs. 5′ and 3′ acceptor stems are shaded green. The D loop 17-nt microhelix is shaded magenta. U-turn stem-loop-stems are shaded yellow (stems) and red (7-nt U-turn loop).

Ribosomes, mRNA, translation systems, genetic coding, and aminoacyl tRNA synthetases (aaRS enzymes; i.e., SerRS) evolved around cloverleaf tRNA. The evolution of tRNA, therefore, is the central problem in understanding evolution of life on earth. A model was determined for the evolution of type-I tRNAs, lacking a V loop expansion (Figure 1) [1,2]. The model is based on the ligation of three 31-nt minihelices followed by two symmetrical 9-nt deletions within ligated 3′- and 5′-acceptor stems. The model posits that type-I and type-II V loops are homologous to acceptor stems.

Cloverleaf tRNA evolved from short, defined genetic segments. A 31-nt minihelix is a 17-nt microhelix flanked 5′- and 3′- by 7-nt acceptor stems. Acceptor stems are based on a GCG repeat and its CGC complement, so the primordial tRNA acceptor stems are 5′-GCGGCGG-3′ (5′-As; As for acceptor stem) and 5′-CCGCCGC-3′ (3′-As). Ligation of a primordial 3′-As and 5′-As, therefore, gives the 14-nt sequence 5′-CCGCCGCGCGGCGG-3′. In generating type-I tRNAs, symmetrical 9-nt deletions leave the sequences 5′-GGCGG-3′ (5′-As*; As* for acceptor stem remnant) and 5′-CCGCC-3′ (3′-As*). The 5′-As* sequence is the last 5-nt of what others describe as the D loop, but which we identify as an acceptor stem remnant [1,2]. The 3′-As* sequence represents the primordial 5-nt V loop sequence for type-I tRNAs.

**Figure 2:**
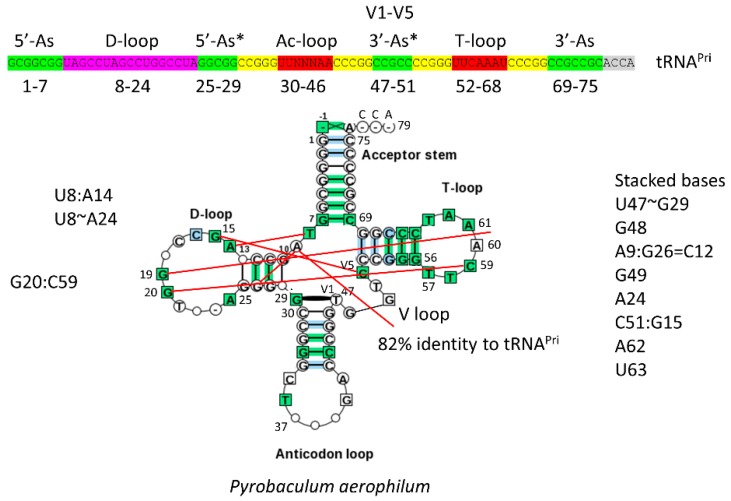
A typical *Pyrobaculum aerophilum* (archaea) tRNA has 82% identity with tRNA^Pri^. Coloring in the schematic (above) is as in Figure 1. Red lines indicate some interactions within the D loop, T loop, and V loop. The typical tRNA has almost two perfect UAGCC repeats (8–17) and identical Ac loop and T loop stems (CCGGG and CCCGG), demonstrating Ac loop and T loop homology.

The 17-nt D-loop microhelix is based on a UAGCC repeat, 5′-UAGCCUAGCCUGGCCUA-3′. The G for A substitution in the third UAGCC repeat allows intercalation of D loop G19 between T loop A60 and A61 in cloverleaf tRNA (Figure 2) [1]. The numbering of tRNAs in this paper follows our adjusted numbering system, based on a D loop that lacks deletions from tRNA^Pri^ (the primordial cloverleaf tRNA). Our numbering, therefore, may vary from what some readers might expect by +3 nt after the D loop (i.e., the anticodon wobble position is listed here as 37 rather than 34).

The structure of the anticodon loop and strong interactions of the D loop, T loop, and V loop make tRNA a relatively stiff and efficient adapter for translation. The anticodon (Ac loop) and T loop microhelices derive from a stem-loop-stem sequence very similar to 5′-CCGGGUUCAAAACCCGG-3′. The CCGGG and CCCGG complementary stems are strongly supported by sequence analysis [1,2]. For tRNA^Pri^, there is slight sequence ambiguity within the 7-nt loops, which, significantly, form a U-turn after the second U (between loop positions 2 and 3). The U-turn within the 7-nt Ac loop is necessary to present a 3-nt anticodon to support a 3-nt genetic code [1]. Without the 7-nt U-turn loop, a 3-nt genetic code would not be possible. In the anticodon loop, loop bases 3–7 stack within the loop as if in a helix, making the 7-nt U-turn Ac loop a compact loop to support a relatively stiff adapter. The T loop has the same 7-nt U-turn loop as the Ac loop, but intercalation of D loop G19 between T loop A60 and A61 lifts A61 to fill the loop, flipping A62 and U63 out of the T loop [1]. Interestingly, A62 and U63 participate in a stack of nucleotide bases that are part of the D loop-V loop-T loop interaction (extending to the “elbow”).

The 3-minihelix model is supported by inspection of archaeal tRNAs from ancient species such as *Pyrococcus furiosis*, *Staphylothermus marinus,* and *Pyrobaculum aerophilum* (Figure 2) [1,3]. There is some controversy in the literature about whether archaea or bacteria are closer relatives to the last universal common ancestor (LUCA), but, in terms of translation systems and tRNA, ancient archaea are clearly most similar to LUCA [4]. This can easily be shown by observing typical tRNA diagrams [3]. Very clearly, the CCGGG and CCCGG Ac loop and T loop stems are conserved, demonstrating that the Ac loop and the T loop are homologs. Because the Ac loop and T loop are homologs, no model based on only two minihelices can account for tRNA evolution [1]. Models based on two minihelices require splitting the Ac stem-loop-stem in two to compare tRNA halves, which is inconsistent with Ac loop and T loop homology, and which is evident from inspection (Figure 2) [1].

The GCG and CGC repeats make up acceptor stems and acceptor stem remnants. The UAGCC repeats in D loop sequences are also apparent. These patterns begin to degrade in bacterial tRNAs with evolution. Significantly, ancient archaeal tRNAs were generated from highly-ordered sequences, repeats, and inverted repeats (i.e., to form stem-loop-stems). Cloverleaf tRNA, therefore, evolved from an ordered and repetitive sequence, identified in some ancient archaea, to a more chaotic sequence in more derived archaea and bacteria.

## 2. Results

### 2.1. A Model for Evolution of Type-II tRNAs

Figure 1 shows a model for evolution of type-I and type-II tRNAs. The model for type-II tRNAs posits that the primordial length of the V loop expansion is 14 nt (7 nt (3′-As) + 7 nt (5′-As)). The model further posits homology of V loops with acceptor stems and acceptor stem remnants. Because archaeal tRNAs are more similar to LUCA tRNAs than are bacterial tRNAs, initially, archaeal tRNAs were collected and compared. In archaea, with rare exceptions, only tRNA^Leu^ and tRNA^Ser^ are type-II tRNAs. We find that expanded and 5-nt V loops are misaligned in tRNAdb and gtRNA databases [3,5,6]. In those databases, V loops were aligned to optimize sequence similarities, introducing inappropriate gaps, rather than, as we align them here, by evolutionary comparisons and secondary structures.

### 2.2. Archaeal tRNAs with Expanded V Loops

Because V loops are variable in length, they are numbered V1 to VN, in which *N* = length of the V loop. For archaeal tRNA^Leu^, *N* = 14, typically, as expected from the model (Figure 1). For archaeal tRNA^Ser^, *N* = 16, typically. Analysis of tRNAomes (all of the tRNAs for an organism displayed as an evolutionary tree and rooted to tRNA^Pri^) indicates that tRNA^Leu^ (*N* = 14) evolves to tRNA^Ser^ (*N* = 16), indicating that V loop expansions are derived from *N* = 14 (Figure 1) [4]. We posit that the initial length of an expanded V loop was *N* = 14, and that longer and shorter V loop expansions are generated by the insertion or deletion of bases most often located approximately to the middle of the V loop.

**Figure 3:**
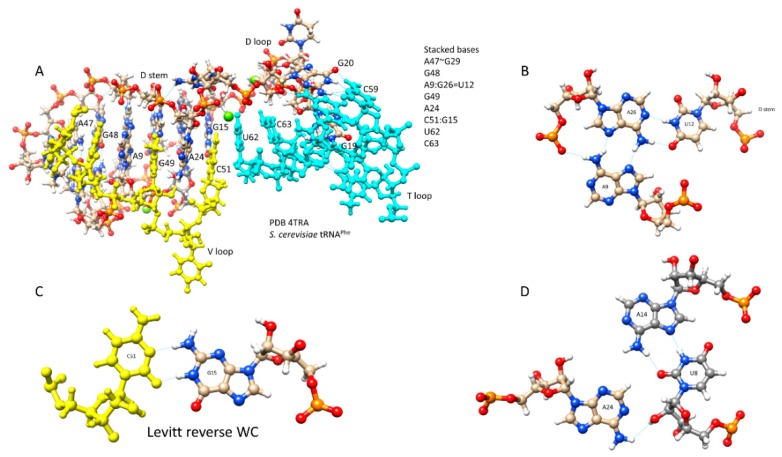
D loop-V loop-T loop interactions (the tRNA “elbow”). (**A**) Stacked bases. (**B**) Interaction of A9-U12-A26. (**C**) The Levitt base pair (G15:C51). (**D**) Interaction of U8-A14-A24. Blue lines indicate hydrogen bonds. The image is from PDB 4TRA [9].

V loops in cloverleaf tRNA are under different selection pressures than acceptor stems. In Figure 3, some of these interactions are highlighted. Figure 3A shows a set of stacked bases stabilizing interactions of the D loop, V loop, and T loop. Figure 3B–D shows some details of interactions. In archaeal tRNA^Leu^ and tRNA^Ser^, U V1 is selected to form a G29~U V1 wobble base pair, and C VN is selected to form a reverse Watson-Crick base pair (G15:C VN), termed the “Levitt” base pair (Figure 3C) [7,8]. In archaea, G15 is often modified to archaeosine, which stabilizes the G15 (archaeosine):C VN interaction, particularly in the presence of Mg^2+^. Typical secondary structures of expanded V loops are selected to be different for tRNA^Leu^ and tRNA^Ser^, so that aaRS enzymes make few errors charging tRNA^Leu^, tRNA^Ser^, and other tRNAs. Similarly, in type-I archaeal tRNAs, a G29~U V1 wobble pair and a G15:C V5 reverse Watson-Crick Levitt base pair are selected strongly.

**Figure 4:**
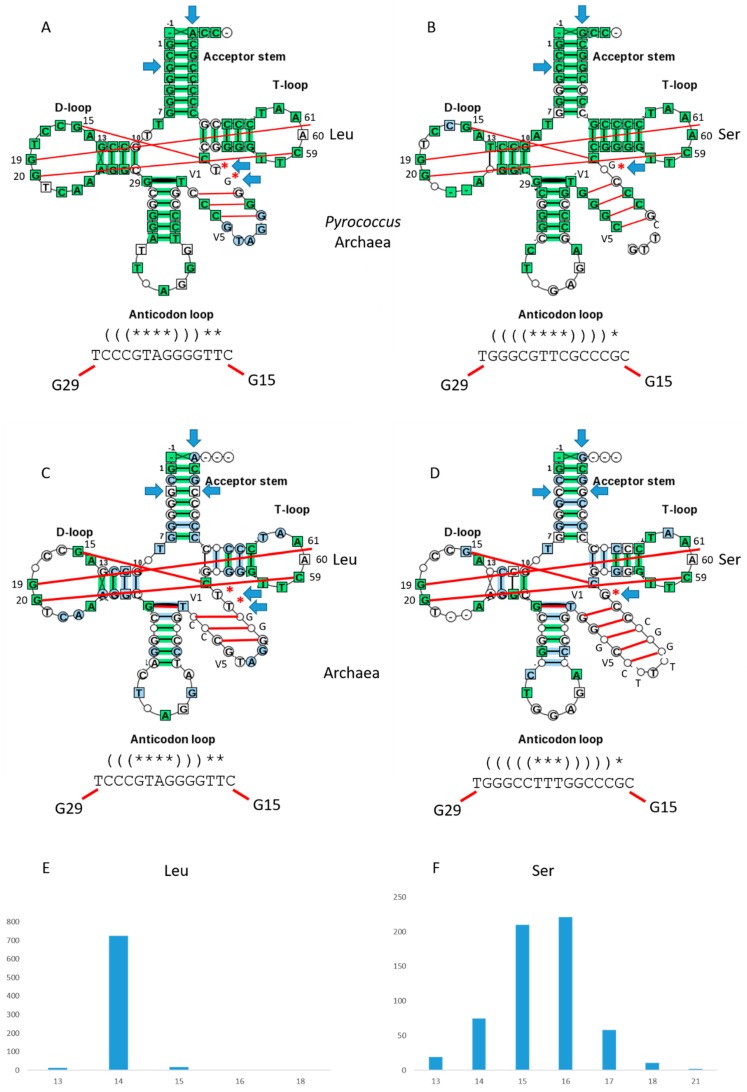
Typical type-II tRNAs in archaea. (**A**) tRNA^Leu^ in *Pyrococcus.* (**B**) tRNA^Ser^ in *Pyrococcus*. (**C**) tRNA^Leu^ in archaea. (**D**) tRNA^Ser^ in archaea. Some interactions within the D loop, V loop and T loop are indicated with red lines. Blue arrows indicate determinants (or anti-determinants) for discrimination of tRNAs by aaRS enzymes. Red asterisks indicate V loop bases not in the V loop stem, that may allow discrimination of different V loops (i.e., by LeuRS, SerRS and other aaRS enzymes). Note that *Pyrococcus* tRNA^Ser^ has two perfect UAGCC repeats in the D loop (8-UAGCCUAGCC-17). (**E**) Histogram of *N* for tRNA^Leu^ in archaea. (**F**) Histogram of *N* for tRNA^Ser^ in archaea.

Figure 4 shows V loop expansions in archaea. As noted above, *Pyrococcus* is an ancient archaeal family with significant similarity to LUCA tRNAs [4]. Three *Pyrococcus* species are compared for tRNA^Leu^ (Figure 4A) and tRNA^Ser^ (Figure 4B). Using typical tRNA diagrams, for tRNA^Leu^, *N* = 14, typically. For tRNA^Ser^, *N* = 15, typically. The G15:C VN Levitt base pair and the G29~U V1 wobble pair are evident. Secondary structures are sufficiently different for LeuRS and SerRS to discriminate tRNA^Leu^ from tRNA^Ser^.

For all archaea, results are very similar (Figure 4C–F). For tRNA^Leu^ (Figure 4C), *N* = 14, typically. For tRNA^Ser^ (Figure 4D), *N* = 16, typically, indicating further V loop expansion through the archaeal domain comparing to the most ancient archaea such as *Pyrococcus* (Figure 4B). Histograms of V loop lengths for archaea are shown in Figure 4E,F. The G15:C VN reverse Levitt base pair and the G29~U V1 wobble pair are evident (Figure 4C,D). Secondary structures are distinct for tRNA^Leu^ and tRNA^Ser^ V loops, so LeuRS, SerRS and other aaRS enzymes can discriminate tRNA^Leu^ and tRNA^Ser^. V loop secondary structures for all archaea are very similar to those observed for *Pyrococcus* tRNAs (Figure 4A,B). As predicted and expected, analysis of archaeal tRNAs with V loop expansions presents a very simple story of evolution that fits to the same model for evolution of type-I tRNAs (Figure 1).

### 2.3. Evolution of Bacterial tRNAs with Expanded V Loops 

**Figure 5:**
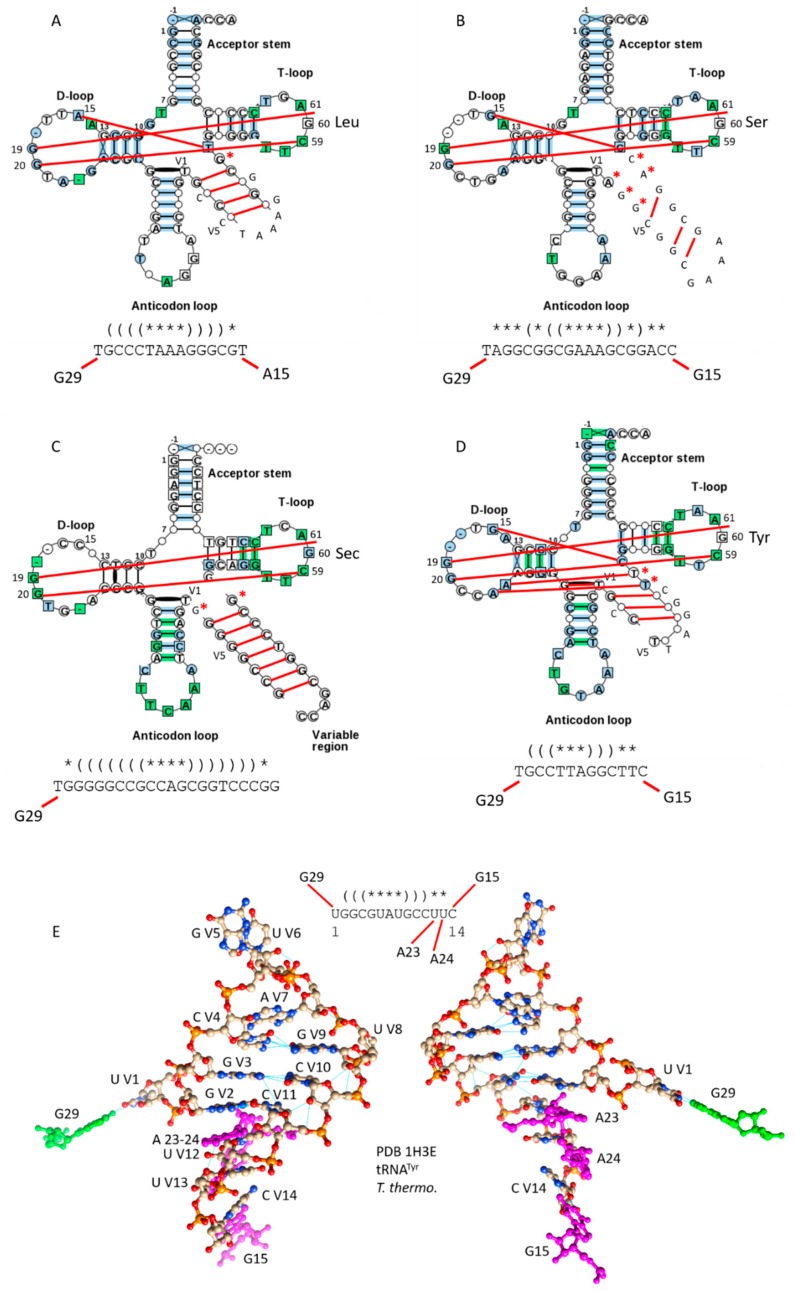
Typical type-II tRNAs in bacteria. (**A**) Bacterial tRNA^Leu^. (**B**) Bacterial tRNA^Ser^. (**C**) tRNA^Sec^. (**D**) tRNA^Tyr^. Some interactions within the D loop, V loop and T loop are indicated with red lines. Red asterisks indicate V loop bases that are not part of the V loop stem, and may allow discrimination of different V loops (i.e., by LeuRS, SerRS and other aaRS enzymes). (**E**) The tRNA^Tyr^ V loop from *Thermus thermophilus* (two views).

Bacteria are expected to be more derived than archaea for tRNA evolution, and bacteria have additional type-II tRNAs that are absent in archaea (Figure 5). In Figure 5A,B, bacterial tRNA^Leu^ and tRNA^Ser^ are compared as typical tRNA diagrams [3]. For bacterial tRNA^Leu^ (Figure 5A), *N* = 15, typically, and for tRNA^Ser^ (Figure 5B), *N* = 19, typically, indicating that bacterial tRNAs are more derived from LUCA than archaeal tRNAs. In contrast to archaeal tRNA^Leu^, in bacterial tRNA^Leu^, an atypical A15: U VN Levitt base pair may be indicated. In bacterial tRNA^Ser^, a conserved G15:C VN Levitt base pair is typical. G29~U V1 wobble pairs are typical for both tRNA^Leu^ and tRNA^Ser^ in bacteria. V loop secondary structures are distinct for archaeal and bacterial tRNA^Leu^ and tRNA^Ser^. The bacterial tRNA^Ser^ V loop is particularly floppy, with fewer stabilizing V loop stem base pairs than are present in archaeal tRNA^Ser^ (Figure 4B,D). The tRNA^Ser^ V loop is a major positive determinant for recognition by SerRS [10]. Because archaeal and bacterial V loop expansions are distinct, it is likely that there is archaeal-bacterial speciation that limits tRNA sharing between domains, i.e., via horizontal gene transfer. Different modifications of tRNAs in the two domains must also suppress tRNA exchanges.

Some bacteria and a few species of archaea utilize tRNA^Sec^ (Sec for selenocysteine) (Figure 5C). To charge tRNA^Sec^, serine is first attached to tRNA^Sec^, and is then modified to selenocysteine [11]. For tRNA^Sec^, *N* = 22, typically, and V loop base pairing is extensive, in contrast to bacterial tRNA^Ser^. The floppiness of the tRNA^Ser^ V loop, therefore, may, in part, help to discriminate tRNA^Ser^ and tRNA^Sec^ to support the tRNA^Sec^ modification pathway. tRNA^Sec^ appears to be derived from tRNA^Ser^. Because tRNA^Sec^ can also be a type-I tRNA, it appears that the ability to process the V loop from type-II to type-I may have persisted in evolution [3,5,6]. Human tRNA^Sec^ (PDB 3A3A) shows a slightly modified cloverleaf fold lacking a Levitt base pair [12].

In bacteria, but not in archaea, tRNA^Tyr^ is a type-II tRNA (Figure 5D). Bacterial tRNA^Tyr^, therefore, may have been reassigned relative to tRNA^Tyr^ in archaea. From analysis of tRNAomes [4], it appears that bacterial tRNA^Tyr^ may have arisen from a tRNA^Ser^. For bacterial tRNA^Tyr^, *N* = 13, typically. The G15:C VN reverse Watson-Crick Levitt base pair is typically present, along with the G29~U V1 wobble base pair. Bacterial tRNA^Tyr^ is discriminated from other bacterial type-II tRNAs by the shorter length of its V loop and the distributions of its V loop stem pairs. In Figure 5E, the structure of the tRNA^Tyr^ 14-nt V loop from *Thermus thermophilus* is shown along with its connections from a co-crystal with TyrRS (PDB 1H3E) [13]. The G15~C V14 and G29~U V1 connections and expected V loop secondary structures are evident. TyrRS reads V loop bases directly to charge tRNA^Tyr^.

### 2.4. Statistical Analyses

#### 2.4.1. Expanded V Loops are Derived from Acceptor Stems

Our model for evolution of type-II tRNA V loops posits that they arose from a sequence very close to CCGCCGCGCGGCGG, which is a primordial 3′-As ligated to a 5′-As (Figure 1). The prediction is tested by comparing the 3′-As to the first 7-nt of V loops and the 5′-As to the last 7-nt of V loops. This approach works for sequences of *N* > 13, so V loops with *N* < 14 were not considered. The first and the last nt were dropped from the comparisons, because the first V loop nt (V1) interacts with position 29 (generally G29~U V1), and the last V loop nt (VN) interacts with position 15 (generally G15~C VN; the Levitt base pair). We are assuming that V loops longer than *N* = 14 have insertions near the center of the sequence.

We have used a random permutation test [1] to indicate the similarities of two long alignments (*n* sequences; *n* is large) of potentially homologous short and aligned sequences. In this comparison, collected alignments of length *l* (*l1* = *l2*) and number of sequences *n* (*n1*, *n2*) are aligned. The code utilized requires comparison of *n1* = *n2* for the two aligned sequences, so, in cases in which *n2* > *n1*, 50 random selections of *n2* = *n1* are selected, and the comparison is repeated. In each comparison, 1000 random permutations of *l2*, *n2* = *n1* are compared to *l1*, *n1*. *P*-values of 50 repetitions are averaged. If the *P*-value for the comparison is small (i.e., <0.05), this indicates similarity and possible homology. If the *P*-value for the comparison is larger (i.e., >0.05), this indicates that sequences are more dissimilar and possibly not homologous. The results are shown in Table 1.

We conclude that expanded type-II V loops are homologous to acceptor stems (3′-As ligated to 5′-As) (Table 1) as predicted by the model for tRNA evolution (Figure 1). Archaeal tRNA^Leu^ and tRNA^Ser^ V loops test as potentially homologous to both archaeal (*P*-values = 0.001 and 0.001) and bacterial (*P*-values = 0.001 and 0.013) acceptor stems. Because 1000 random permutations are compared, the lowest *P*-value that is possible is 0.001. This result appears to demonstrate the model shown in Figure 1 for V loop expansions. The bacterial tRNA^Leu^ V loop also tests as potentially homologous to archaeal acceptor stems (*P*-value = 0.020). Bacterial tRNA^Sec^ (Sec for selenocysteine) tests as being likely homologous to archaeal acceptor stems (*P*-value = 0.001). Interestingly, bacterial tRNA^Tyr^ with an expanded V loop tests as potentially homologous to bacterial acceptor stems (*P*-value = 0.020) but not to archaeal acceptor stems (*P*-value = 1). The bacterial tRNA^Tyr^ *P*-value = 0.020 result may be attributable to convergent evolution of C-G rich sequences, because bacterial tRNA^Tyr^ is probably derived from sequences similar to bacterial tRNA^Leu^ or bacterial tRNA^Ser^. Also, similarly to bacterial tRNA^Tyr^, bacterial tRNA^Ser^ scores as more similar to bacterial acceptor stems (*P*-value = 0.277) than archaeal acceptor stems (*P*-value = 0.999). As we have shown in other ways, archaeal tRNAs are significantly less radiated from a primordial tRNA than bacterial tRNAs [1,4], and this observation is documented from comparisons of type-II tRNA V loop expansions.

#### 2.4.2. Kinship of Expanded V Loops

**Figure 6:**
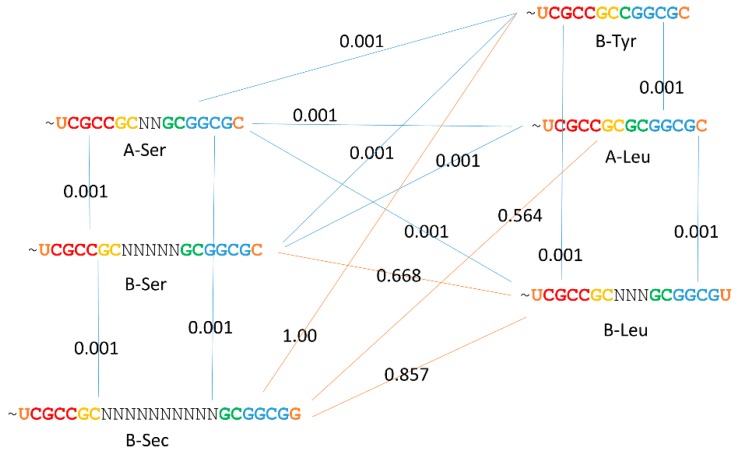
Relatedness of V loop expansions. *P*-values are shown. Blue lines indicate detected homology. Orange lines indicate V loop divergence (i.e., for aaRS discrimination). “A” indicates archaea. “B” indicates bacteria.

The relatedness of expanded V loops is described in Figure 6 using comparisons of *P*-values. Once again, a *P*-value < 0.05 indicates probable homology of compared alignments. Archaeal tRNA^Leu^ and tRNA^Ser^ are closely related in sequence [4], and all bacterial V loop expansions appear to relate closely to archaeal tRNA^Leu^ and tRNA^Ser^. These results indicate that archaeal tRNAs are closer to LUCA tRNAs than bacterial tRNAs. In bacteria, tRNA^Leu^ and tRNA^Ser^ have diverged from one another, presumably to support aaRS discrimination in tRNA charging [10]. We posit that type-II bacterial tRNA^Tyr^ was derived from tRNA^Ser^ through a process of tRNA re-assignment, which we have previously described for another tRNA [4]. Essentially, a type-I tRNA^Tyr^ (as in archaea) was eliminated and replaced by a type-II tRNA^Tyr^ derived from a tRNA^Ser^.

## 3. Discussion

The mechanism for type-II tRNA evolution is the same as the mechanism for type-I tRNA evolution, but lacks a processing step (Figure 1). The primordial length of the type-II V loop was 14-nt, as observed for archaeal tRNA^Leu^ (Figure 4). We posit that tRNA^Leu^ evolved to tRNA^Ser^, which has a longer V loop because of insertions near the middle of the loop [4]. Both in length and sequence, archaeal tRNA^Leu^ and tRNA^Ser^ appear closer to LUCA tRNAs than bacterial tRNA^Leu^ and tRNA^Ser^. Type-II bacterial tRNA^Tyr^ and tRNA^Sec^ appear to be derived from tRNA^Ser^. Bacterial type-II tRNA^Tyr^ appears to have evolved by tRNA reassignment: elimination of a type-I tRNA^Tyr^ and reassignment of a tRNA^Ser^ to tRNA^Tyr^.

The model for evolution of type-I tRNAs was developed by inspection, similarly to the solving of a puzzle [1,2]. From inspection of typical tRNA diagrams, using ancient archaea, homology of the Ac and T stem-loop-stems was evident (Figure 2). This accounts for 34-nt of tRNA, and the type-I tRNA core (lacking 3′-ACCA) is initially 75 nt. Considering acceptor stems, 48 nt of tRNA are described. This is more than half the tRNA. Eventually, the D loop microhelix was solved as a truncated UAGCC repeat of 17 nt (Figure 2). Because three 17-nt microhelix sequences were present (D loop, Ac loop and T loop), and the D loop and T loop were flanked on one side by a 7-nt acceptor stem, this indicated that the molecule was derived from three 31-nt minihelices. Therefore, evolution of type-I tRNAs could be solved by two symmetrical 9-nt deletions, and the two remaining 5-nt sequences were the last 5 nt of the D loop (5′-As*) and the 5-nt V loop (3′-As*) (Figure 1). The length of the primordial cloverleaf tRNA lacking 3′-ACCA was 75 nt (Figure 1 and Figure 2). This model for type-I tRNA evolution was strongly supported using a battery of statistical sequence comparisons [1]. In the current paper, the model for type-I tRNA evolution was extended to describe evolution of type-II tRNAs with V-loop expansions (Figure 1). The model for type-II tRNAs is identical, except there is no processing of the 3′ 14-nt ligated 3′- and 5′-acceptor stems (7-nt + 7-nt), which becomes the expanded V loop. Slight variations of the same model, therefore, completely and cleanly account for evolution of type-I and type-II tRNAs.

Type-II tRNAs have been processed in evolution to type-I tRNAs, consistent with our model for evolution of type-I tRNAs (Figure 1). Notably, tRNA^Ser^ and tRNA^Sec^, which primarily are type-II tRNAs, include type-I tRNAs [3]. The model in Figure 1 indicates that the first cloverleaf tRNAs may have included 93, 84 and 75-nt core sequences. Most tRNAs are of the type-I variety, but tRNA^Leu^, tRNA^Ser^, tRNA^Sec^ and bacterial tRNA^Tyr^ remained mostly type II. Because of the apparent appearance of type-I tRNAs from type-II tRNAs in modern lineages, it is possible that type-II tRNAs can still be processed under some conditions to type-I tRNAs. Perhaps, these 93, 84, and 75-nt species of tRNA cloverleafs co-existed, until selection of primarily type-I tRNAs and a smaller collection of type-II tRNAs. It appears that V loop lengths and sequences were mostly selected to optimize discrimination by ~20 aaRS enzymes [10].

### 3.1. Comparison of tRNA Evolution Models

Competing models for tRNA evolution have been advanced [14,15,16,17,18,19]. Some of these models indicate that two minihelices might be ligated to form a primordial tRNA. Our model, by contrast, requires ligation of three 31-nt minihelices representing two different 17-nt microhelix core sequences (1:2; the D loop and the homologous Ac and T stem-loop-stems) (Figure 1). We show clearly that the Ac loop and T loop are homologs (Figure 2) [1]. In a two minihelix model, however, the Ac and the T stem-loop-stem cannot be homologous, because the Ac loop must be bisected to make the comparison, spoiling the alignment. Rather, a two minihelix model predicts that the D loop and the T loop should be similar in sequence, which they clearly are not. As we show here, and as we have shown previously, the D-loop microhelix is based on a UAGCC repeat, which cannot be similar in sequence to a CCGGGUUCAAAUCCCGG T stem-loop-stem [1]. In Figure 4B, we show two perfect UAGCC repeats in the D loop, indicating the UAGCC repeat. Another criticism of the two minihelix models is that they appear to require unlikely sequence and structural convergence of the 7-nt U-turn Ac and T loops. If the homology of the Ac and T stem-loop-stems is accepted (Figure 2), only the three minihelix model makes sense. One proposed two minihelix model is based too heavily on analysis of tRNA introns in the Ac loop of one archaeal species [15]. Introns are found in many sites of archaeal tRNAs, not just in the Ac loop [20]. Our three minihelix model is strongly supported by identification of internal D loop (5′-As*) and V loop (type I: 3′-As*; type II: 3′-As ligated to a 5′-As) homologies to acceptor stems. Our model for type-II tRNAs strongly supports the model we previously proposed for processing a ligated 3′-As and 5′-As (14 nt) by deletion of 9 nt to yield a 3′-As* type-I V loop [1], because we identify the previously predicted intermediate in processing to a type-I tRNA as existing in type-II tRNA. Put more simply, type-II tRNA is the predicted intermediate in processing of type-I tRNA (Figure 1) [1]. Our model makes strong sequence predictions, which are all justified by statistical tests (Table 1) [1]. So far as we can judge, two minihelix models do not make strong sequence predictions that can be justified by any analysis we can apply.

### 3.2. Evolution of the Genetic Code

Because tRNA evolution is such a simple story, evolution of the genetic code and translation systems becomes simpler to understand [4,21]. Significantly, the tRNA-centric view provides a simplified understanding of genetic code evolution. As viewed from the perspective of mRNA, in which all 64 codons are used, >10^84^ genetic codes and up to 63 encoded amino acids might be possible [22]. Viewed from the perspective of tRNA, however, the genetic code is half the size: a 32-letter code in tRNA versus a 64 letter code in mRNA [4,21]. The reason the code in tRNA is smaller than it is in mRNA is that ambiguity in reading the wobble position of tRNA limits the size of the code. Essentially, because codon-anticodon contacts are not fully proofread for the wobble position base on the ribosome, there is only purine versus pyrimidine discrimination at the wobble position, not single base (A,G,C,U) recognition. The single exception is tRNA^Ile^ (UAU) versus tRNA^Met^ (CAU), which is supported by extensive modifications to tRNA^Met^ (CAU) [23,24,25]. Furthermore, for the most part, tRNA^Ile^ (UAU) is only utilized in eukaryotes and not in prokaryotes. The maximum complexity of the genetic code in tRNA, therefore, is 4 × 4 × 2, instead of 4 × 4 × 4 in mRNA. The standard code, therefore, evolved to encode 20 amino acids rather than a larger number. There are additional dimensions to this story described in other work [4,21].

### 3.3. Evolution of tRNA Sequence Proceeded from Order to Chaos

Archaeal tRNAs are better preserved from LUCA than bacterial tRNAs, and archaeal tRNAs are more highly-ordered in their sequence [1,2]. Ancient archaea such as *Pyrococcus*, *Pyrobaculum* and *Staphylothermus* have tRNAomes (i.e., typical tRNA diagrams) that are more similar to a LUCA tRNAome than more derived species [4]. These tRNAomes are more ordered in sequence, because tRNA evolved from repeating sequences (Figure 1). The ancient world of ~4 billion years ago, therefore, in some cases, evolved biological complexity from ordered sequence, in the form of repeats and inverted repeats. From analysis of tRNA evolution, therefore, the assumption that biological complexity was generated only from random polymer sequences is incorrect. Processes such as replication slippage and abortive initiation generated repeats and/or short RNA fragments that could be attached by ligation. Evolution from ordered repeats to chaos can clearly be seen in tRNA evolution, by inspection of typical tRNA diagrams for ancient archaea compared to more derived bacteria. Furthermore, mechanisms probably existed in the ancient world to measure the lengths of sequences, because repeats were clipped into functional units of 5 nt (Ac and T loop stems), 7 nt (i.e., acceptor stems, Ac and T loops) and 17 nt (i.e., D loop, Ac loop and T loop microhelices). Of course, these length selections may represent selections for evolving biological function.

### 3.4. The Inanimate to Animate Transition

**Figure 7:**
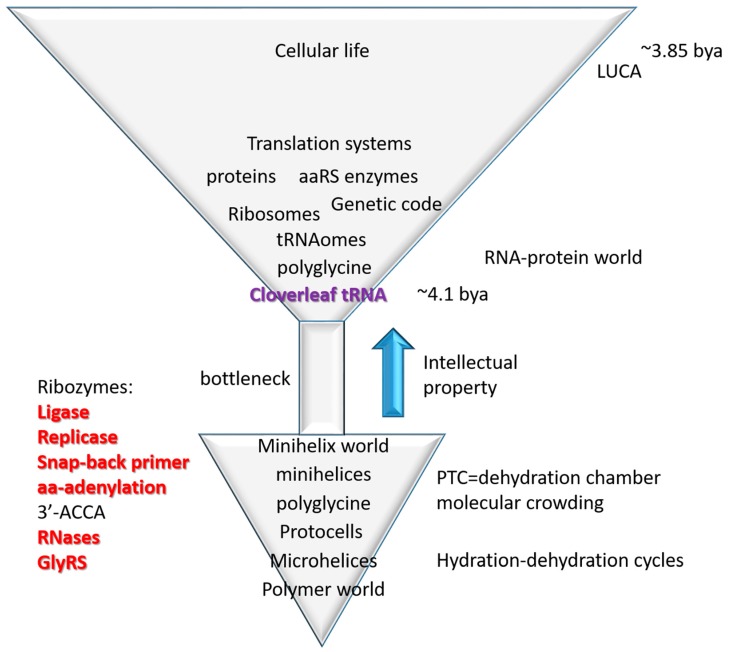
Evolution of translation systems. A simple model for the inanimate→animate transition based on the evolution of cloverleaf tRNA from sequence repeats and 31-nt minihelices including stem-loop-stems (see Figure 1). See the text for details.

The central advance in biological intellectual property in evolution of life on earth was cloverleaf tRNA, the adapter that permits biological coding, and around which coding functions evolved [4,21]. From conserved tRNA sequences, the pathway of tRNA evolution has been determined (Figure 1). This is a story of building biological complexity from ordered repeats and snap back stem-loop-stems. So, life on earth was snapped together (ligated) similarly to the children′s game of LEGO (trademark) (Figure 1). The inanimate to animate transition is described as a simple model in Figure 7, tracking the evolution of microhelices→minihelices→cloverleaf tRNA→translation systems→cellular life. Life, therefore, evolved from a primitive inanimate polymer world that includes short sequences (i.e., ACCA; abortive initiation), repeats (i.e., GCG, CGC and UAGCC repeats; replication slippage) and inverted repeats (Ac loop and T loop microhelices; stem-loop-stems, which can attach to form replication primers). Polymers are generated via dehydration reactions, so cycles of hydration and dehydration may be sufficient to describe generation of the first biopolymers [26,27].

From a strange polymer world that includes 17-nt microhelices (i.e., D loop, Ac loop and T loop microhelices), a small collection of ribozymes appears necessary and possibly sufficient to generate cloverleaf tRNA and translation systems. These ribozyme activities have been largely reinvented in vitro, and some of these ribozymes can be quite small, indicating that their evolution via simple non-biotic processes might be possible [28,29,30,31,32,33,34]. Hydration-dehydration cycles drive polymerization reactions and concentrate cofactors such as Mg^2+^.

As we have previously proposed, microhelices, minihelices, and cloverleaf tRNA may have been initially evolved to synthesize polyglycine, proposed to have been used to stabilize protocells, as in bacterial cell walls [4,21]. We note that polyglycine requires a membrane anchored carbohydrate to form cell wall-like cross links, so polyglycine by itself is not sufficient for stabilization of protocells. One evidence for the polyglycine model is that tRNA^Pri^ (the primordial tRNA cloverleaf; Figure 2) is closest in sequence to tRNA^Gly^ in ancient archaea [4]. We have described a Darwinian pathway to evolve the 21-letter genetic code (20 amino acids with stops) from a one letter code synthesizing polyglycine (using any mRNA sequence). Once cloverleaf tRNA evolves, therefore, evolution of the genetic code appears to be assured. We conclude that evolution of the tRNA cloverleaf is the major advance in evolution of biological intellectual property that led to evolution of the genetic code, translation systems and cellular life. Once cloverleaf tRNA evolves, Darwinian selection drives evolution of dependent processes (translation, genetic code, aaRS enzymes), so evolution of cloverleaf tRNA is the core advance.

As described previously, the evolution of the ribosome requires initially a scaffold on which to mount and move mRNA (a decoding center) and perhaps a mobile peptidyl transferase center [2]. The peptidyl transferase center can be viewed as a dehydration and molecular crowding chamber to drive the polymerization of polypeptide chains [27]. If the peptidyl transferase center is a “ribozyme”, it is not a good one. Every other function of the ribosome is a refinement or add-on: i.e., translocation, proofreading, initiation, and termination functions. Unlike tRNA (Figure 1), the evolutionary source of rRNA is more obscure. Without breakthrough success, our laboratory has attempted to solve this problem in collaboration with Robert Root-Bernstein (MSU). As a cautionary tale, rRNA sequences appear cloverleaf tRNA-like, and ancient archaeal rRNAs appear more tRNA-like than more derived species, indicating that cloverleaf tRNA was one of the building blocks of rRNA sequences. Back translating rRNA sequences (BlastX; NCBI) gives apparent open reading frames, but none of these can clearly be traced to an independent functional gene or close homolog. Generally, in archaeal and bacterial genomes, these long open reading frames are only found in rRNA sequences. Open reading frames with an apparent annotation, i.e., a “cell wall hydrolase” embedded in the peptidyl transferase center of 23S rRNA as a reverse orientation gene, cannot be confirmed to encode a cell wall hydrolase with a known function that exists anywhere as a stand-alone gene or that has notable homology to other cell wall hydrolases or structures. We concluded that this sequence does not encode a cell wall hydrolase, and was wrongly annotated but propagated through the reported annotations of many genomes leading to potential confusion, including our own. So, although a fairly simple model for ribosome evolution can be proposed, to our knowledge, in contrast to tRNA (Figure 1), the detailed evolution of rRNA sequences and the evolution of the ribosome remain largely unsolved but compelling mysteries.

## 4. Materials and Methods 

Methods and databases have been described previously [1,2,3,4,21]. The statistical permutation test is useful for comparing two short, aligned sequences with many examples of each for possible homology [1]. Most tRNA sequences were taken from gtRNAdb [5,6]. For convenience, typical tRNA diagrams were taken from the older tRNAdb [3]. V loop sequences were added to typical tRNA diagrams by hand. Structures were analyzed using UCSF Chimera [35,36].

## 5. Conclusions

We conclude that evolution of cloverleaf tRNA, as described here (Figure 1), drove the inanimate to animate transition in evolution of life on earth (Figure 7). The inanimate world is characterized by a strange polymer world with unexpected order that includes sequence repeats (i.e., GCG, CGC and UAGCC repeats), short abundant potentially functional sequences (i.e., ACCA), and snap-back primers (stem-loop-stems, inverted repeats; microhelices, minihelices). Ordered polymers led to evolution of microhelices, minihelices, and cloverleaf tRNA (Figure 1). Once cloverleaf tRNA evolved, evolution of the genetic code, the ribosome, translation systems, and cellular life were assured. Strangely, very few uncertainties remain in this amazing story recorded and told in genetic sequences that evolved about 4 billion years ago.

## Figures and Tables

**Table 1:** Homology of type-II V loops to acceptor stems (Arch: archaea; Bact: bacteria).

	***P*-values against Archaeal Acceptor Stems**					
**V loop**	Arch LEU	Arch SER	Bact LEU	Bact SER	Bact TYR	Bact SEC
**AVERAGE**	0.001	0.001	0.020	0.999	1.000	0.001
	***P*-values against Bacterial Acceptor Stems**					
**V loop**	Arch LEU	Arch SER	Bact LEU	Bact SER	Bact TYR	Bact SEC
**AVERAGE**	0.001	0.013	1.000	0.277	0.020	0.860

## Abbreviations

aaRS	Aminoacyl-tRNA synthetase (i.e., LeuRS)
As	Acceptor stems
As*	Acceptor stem remnants
Ac loop	Anticodon loop
LUCA	Last universal common (cellular) ancestor
T loop	T loop or TΨC loop
V loop	Variable loop
